# Intelligent Rehabilitation Assistance Tools for Distal Radius Fracture: A Systematic Review Based on Literatures and Mobile Application Stores

**DOI:** 10.1155/2020/7613569

**Published:** 2020-09-29

**Authors:** Yalan Chen, Yijun Yu, Xin Lin, Zhenwei Han, Zhe Feng, Xinyi Hua, Dongliang Chen, Xiaotao Xu, Yuanpeng Zhang, Guheng Wang

**Affiliations:** ^1^Department of Medical Informatics, School of Medicine, Nantong University, Nantong 226001, China; ^2^Department of Hand Surgery, Affiliated Hospital of Nantong University, Nantong 226001, China; ^3^Nantong University Xinglin College, Nantong University, Nantong 226236, China; ^4^Bachelor of Nursing, University of Technology Sydney, Sydney, NSW 2007, Australia

## Abstract

**Objective:**

To systematically analyze the existing intelligent rehabilitation mobile applications (APPs) related to distal radius fracture (DRF) and evaluate their features and characteristics, so as to help doctors and patients to make evidence-based choice for appropriate intelligent-assisted rehabilitation.

**Methods:**

Literatures which in regard to the intelligent rehabilitation tools of DRF were systematic retrieved from the PubMed, the Cochrane library, Wan Fang, and VIP Data. The effective APPs were systematically screened out through the APP markets of iOS and Android mobile platform, and the functional characteristics of different APPs were evaluated and analyzed.

**Results:**

A total of 8 literatures and 31 APPs were included, which were divided into four categories: intelligent intervention, angle measurement, intelligent monitoring, and auxiliary rehabilitation games. These APPs provide support for the patients' home rehabilitation guidance and training and make up for the high cost and space limitations of traditional rehabilitation methods. The intelligent intervention category has the largest download ratio in the APP market. Angle measurement tools help DRF patients to measure the joint angle autonomously to judge the degree of rehabilitation, which is the most concentrated type of literature research. Some of the APPs and tools have obtained good clinical verification. However, due to the restrictions of cost, geographic authority, and applicable population, a large number of APPs still lack effective evidence to support popularization.

**Conclusion:**

Patients with DRF could draw support from different kinds of APPs in order to fulfill personal need and promote self-management. Intelligent rehabilitation APPs play a positive role in the rehabilitation of patients, but the acceptance of the utilization for intelligent rehabilitation APPs is relatively low, which might need follow-up research to address the conundrum.

## 1. Introduction

Distal radius fracture (DRF) is one of the common upper limb fractures, which could be accounted for about 15.7% of upper limb fractures [1]. It is more common in elderly women; this may be due to the effects of osteoporosis and eventually lead to falls. Due to the influence of osteoporosis and the increase of age, slight external force could often result in complex comminuted fracture, and with the possible injury of intercarpal ligament for postmenopausal women.

Majority of the patients with this type of fracture are mainly middle-aged and elderly women, most of whom still have a misunderstanding that they need to rest and cannot exercise during the recovery phase. Exercise intervention has been proven to be one of the most effective ways to improve health and promote recovery [2]. Appropriate rehabilitation exercise can accelerate local blood circulation; accelerate the repair of surrounding soft tissues; prevent muscle atrophy, joint cavity adhesion, and stiffness; and reduce the occurrence of complications such as dysfunction of joints near the fracture site [3].

Elderly patients with fractures have poor compliance with postoperative rehabilitation training. In addition, in the short-term hospitalization period, it is difficult for patients to master the comprehensive knowledge and skills of rehabilitation training and to guarantee the progress and quality of independent rehabilitation training after discharge. Moreover, some traditional rehabilitation methods require patients to conduct at a fixed time and place, and some of the method is relatively boring. Too much repetitive exercise makes it difficult for patients to persist for a long time and lack motivation.

mHealth is a medical and public health practice supported by mobile devices that fills the above gap and promotes unprecedented opportunities for professional clinical diagnosis and treatment recommendations [4, 5]. As a software application accompanying mHealth, commonly known as “application” (APP), it has become one of the main ways for patients to participate in health and rehabilitation management. It can realize home rehabilitation training for patients, self-diagnose the rehabilitation progress, and obtain professional rehabilitation guidance, which greatly promotes the development of participatory medicine [6, 7]. For example, the Serious Game APP could be regarded as effective mean for intervention in home exercise projects [8], while the protractor software could measure the angle of arm rotation and abduction for patients and evaluate the degree of recovery by collecting each measurement data.

At present, the practical utilization of intelligent rehabilitation assistance tools related to DRF is not very extensive. In many cases, patients find it difficult to choose appropriate tools according to the rehabilitation needs when they are faced with a variety of APPs on different mobile platforms. Therefore, we summarize the up-to-date APPs related to DRF in both literatures and different mobile platforms. Through evidence-based retrieval and data extraction, we systematically analyze the functions, characteristics, and limitations of these APPs, to provide evidence-based references for clinical and patients.

## 2. Materials and Methods

### 2.1. Data Retrieval

A systematic review of the literature was performed by searching PubMed, the Cochrane Library, Wan Fang Data, and VIP Data for relevant literatures on February 20, 2020. The selected research is mainly about smart or intelligent APPs related to the rehabilitation of DRF patients. Based on the relative review studies and discussions with hand surgeons, as well as adjustments in the actual retrieval process, the following retrieval strategies were finally formulated: (distal radius fracture [tiab] OR DRF [tiab] OR fractur∗ [tiab])AND (smartphone [tiab] OR application [tiab] OR app∗ [tiab] OR iphone [tiab]) NOT (Amyloid precursor protein [tiab]).

The intelligent rehabilitation tools on the APP market are mainly divided into several categories such as health education and functional exercise. “distal radius fracture”, “rehabilitation”, “functional exercise” or “health education” were searched in Chinese and English separately in APP store, Google player, and Huawei platform.

### 2.2. Inclusion and Exclusion Criteria

Inclusion criteria include the following: (1) the study about the intelligent rehabilitation APPs or tools for the DRF patient, especially intervention research; (2) APPs or tools that assist the intelligent rehabilitation for the DRF patient on any platform; (3) rehabilitation is the main function or included function of the APPs or tools; and (4) the APP was applicable from intelligent mobile terminals.

Exclusion criteria include the following: (1) APPs or tools limited to the operation and use of medical staff, (2) APPs with no score or score less than 3 and download volume less than 100, (3) APPs or tools for rehabilitation of other fracture types, and (4) APPs or tools without detailed description and usage records.

### 2.3. Study Selection and Data Extraction

Data from the included studies were extracted by the two investigators (J.Y. and D.C.) using standardized and piloted design formats. The preset extraction indicators mainly include basic information such as the name of the APP, the platform used, and the function. The literature research also needs to extract the clinical research methods and results, and the platform information needs to include user usage, downloads, and ratings. Discrepancies in the process of study selection and data extraction were resolved through a group discussion with two other authors (Y.C. and G.W.).

The extraction of the intelligent rehabilitation APPs involved in the literature, classified them according to the functions and types of different APPs, and analyzed the advantages and disadvantages.

## 3. Results

### 3.1. Eligible Studies and Characteristics

**Figure 1:**
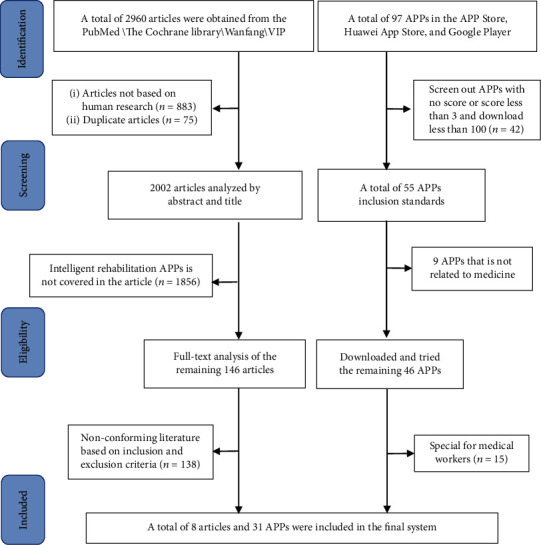
The selection process of literatures and APP stores.

The flow of the search strategy is shown in Figure 1. A total of 2960 articles were searched in the four databases. Subsequently, 883 non-human research articles were screened out through sifting and screening. The remaining 2002 articles were analyzed by abstract and title, then 146 for full-text analysis, and 8 articles were finally included (Table 1). Ninety-seven intelligent rehabilitation APPs were sifted through according to the description by the publisher and the rating. Finally, 31 APPs related to the rehabilitation of DRF were included (Supplementary Table 1).

### 3.2. Brief Analysis of Intelligent Rehabilitation Tools

**Figure 2:**
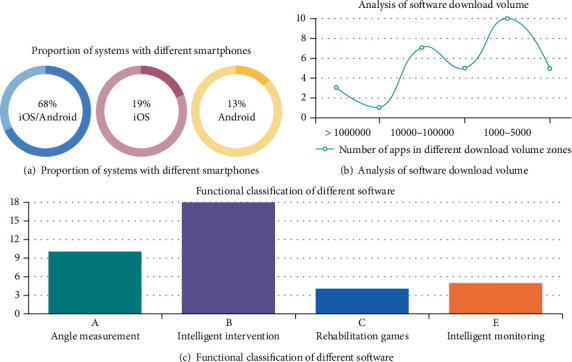
Brief analysis of intelligent rehabilitation tools.

It can be seen from the result that 25 (68%) smart APPs are applicable to both iOS and Android, with 7 (19%) smart APPs are suitable for iOS system, and 5 (13%) smart APPs are suitable for Android system (Figure 2(a)). Most of the APPs are free.

### 3.3. Analysis of Downloads and User Evaluations of Different APPs

The number of APP downloads from the APP market is concisely described in Figure 2(b). The top three APPs with more than 100,000 downloads are the intelligent intervention APP “Fisioterapia a tu alcance” and two intelligent monitoring APPs “OASIS Healthcare (HongHua Medical)” and “Gold nurse (gold nurse).” The three software not only have the highest downloads but also got a user score higher than 4 points, which was highly praised by users.

### 3.4. Classification and Function Characteristics of Different APPs

Intelligent rehabilitation APPs were divided into five categories according to different functions: intelligent intervention, angle measurement, intelligent monitoring, and rehabilitation game [17] (Figure 2(c)).

Intelligent intervention software was accounted for the most of the results retrieved in the APP market. Intelligent intervention is mainly used in the later stage of fracture, which is a critical period for functional exercise after reaching the clinical healing standard. Such APPs would be able to provide patients with functional exercise and develop personalized rehabilitation services by combining physiotherapy (PT) and home exercise programs (Heps).

**Figure 3:**
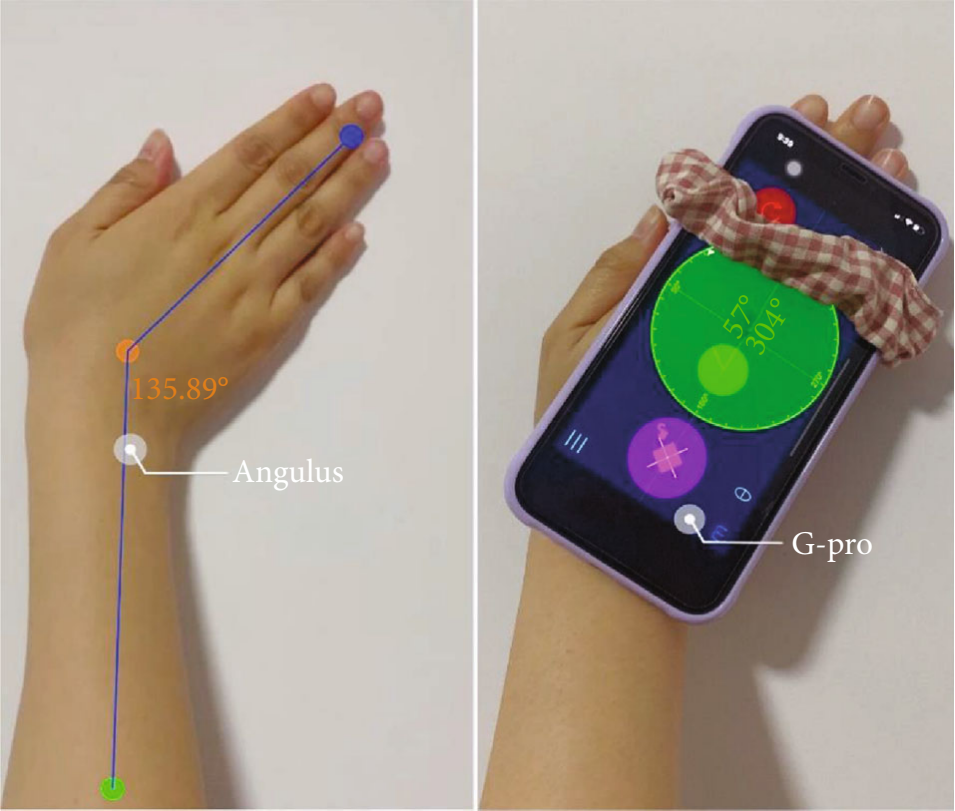
The ulnar deviation measurement demo of the Anglus and G-Prp.

The second proportion of the results retrieved in the APP market is the angle measurement APP which holds the largest proportion among selected literatures regarding intelligent rehabilitation APPs. The degree of rehabilitation of DRF patients is generally diagnosed by the angle of extension, flexion, pronation, and supination of the wrist and forearm. The angle measurement tool allows the patient to measure and record the rehabilitation angle without leaving home and can make independent judgments or share the data with the doctor in real time so that the doctor can understand the degree of the patient's rehabilitation. The ulnar deviation measurement of the two APPs is demonstrated in Figure 3.

There are not many rehabilitation games related to fractures in the APP store and literature studies. The way of games is conducive to divert the patient's attention, in the process of playing games to enhance wrist finger movement and joint training and reduce the discomfort in the training process.

**Figure 4:**
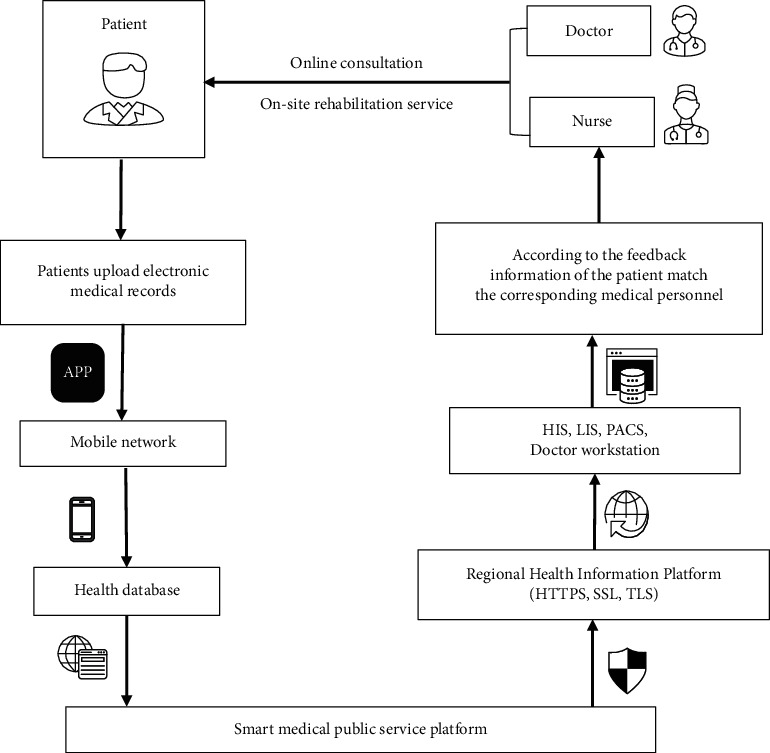
Briefly introduced the intelligent monitoring APPs. HIS: hospital information system; LIS: laboratory information management system; PACS: picture archiving and communication systems; HTTPS: Hypertext Transfer Protocol over Secure Socket Layer; SSL: Secure Sockets Layer; TLS: Transport Layer Security.

The intelligent monitoring APP involves more complicated processes and links, providing patients with an emerging medical service model (Figure 4). It can combine online consultation with offline care to provide remote services for patients, ease the gathering and intersection of outpatient populations, promote closer communication between doctors and patients, and improve rehabilitation effects.

## 4. Discussion

The popularization of mHealth technology has gradually brought a new experience and service mode to the public, which would help to address the disadvantages of the existing medical system [18, 19]. This research combines scientific research with the practical APP market, comprehensively analyzes the current status of the utilization of DRF's intelligent rehabilitation APP, and discusses the characteristics of different types of rehabilitation APPs.

From the research results of the APP market, the most involved are intelligent intervention APPs, the average downloads are over 1000. The most popular APP is the intelligent monitoring APP, which scores more than 4 points. In contrast, the most involved in literatures are the angle measurement APPs, followed by rehabilitation game APPs. This may indicate that angular measurement and rehabilitation game APPs have more research value; meanwhile, intelligent intervention and intelligent monitoring are more valuable regarding commercial and economic areas. The APP involved in some literatures does not yet exist in the APP market, which may indicate that some APPs are still in the development stage. However, most of the APPs in the APP market lack effective evidence and real data support, and the actual medical value needs further verification.

### 4.1. The Advantages and Disadvantages of Different Types of Rehabilitation APPs

Studies have shown that the prognosis of patient with DRF would benefit from the combination of supervised PT and Heps [20]. Intelligent intervention APPs realize the combination of the two methods. On one hand, the real-time transmission of smart phone devices realizes the connection between patients and medical service providers, which is conducive to the development of personalized guidance programs [21]. Meanwhile, these APPs also provide professional rehabilitation exercise guidance which promotes correct functional exercise [22]. On the other hand, intelligence software runs in the background to supervise patients to complete corresponding functional exercises and provide users with timely feedback through charts and other forms of information to improve patients' emotional and psychological conditions in time [23].

Most of the patients with DRF would self-diagnose their condition and recovery process by judging proprioceptive recovery such as grip strength, pinch force, and wrist movement. Few patients would determine their rehabilitation process by measuring the angle of wrist movement [24, 25]. Nevertheless, the current understanding and knowledge in regard to wrist proprioception is still not enough [26–28]. Distal fractures of the radius might often involve with the wrist [29, 30], which would limit the range of movement (ROM) between the forearm and wrist in multiple motor planes, including wrist flexion and extension, carpal ulnar deflection, and forearm pronation [31, 32]. Hence, the ROM would be more accurate when determining the recovery process of DRF [11]. The angle measurement involved in this article mainly measures the range of motion and angle in two ways. For example, the measurement of ulnar deviation, one is to upload the angle photo or video of the part to be measured to the APP and then measure based on the image data; the other is to fix the mobile device on the part to be measured on the wrist or forearm and measure the movement amplitude (angle) in real time (Figure 3). The accuracy and precision of these two methods are affected by the accuracy of the method used by the patients.

Patients with DRF usually experience symptoms such as limited wrist movement [33], pain, weakness, and even serious complications (nonunion [34] and malunion [35]). Professional rehabilitation care is beneficial for pain management and reduction of the occurrence of adverse complications [36]. Intelligent monitoring APP helps professional medical staff and patients to establish harmonious relationship [10], provide timely and professional medical services, and reduce the occurrence of adverse complications. Moreover, it can provide remote consultation and home care. Compared with the traditional medical model, it can greatly save patients' waiting time and improve patient satisfaction and rehabilitation flexibility.

The advent of the Internet era would allow non-text-based interactive information, such as video and images, to become more acceptable [37]. Rehabilitation games are interactive APPs that are based on the internet and visual stimulation which could also combine with wearable sensors. For example, “ReValidate!” [14] is an APP that needs to be used in conjunction with wearable devices on the proximal end of the wrist and forearm. By monitoring the parameters of the patient's wrist or upper limb motion range [32], it can safely and effectively help the DRF patient's wrist rehabilitation [8]. However, the use of these APPs is affected by the patient's cognition and acceptance and requires the guidance of professionals. At the same time, due to cost and geographic restrictions, the promotion of some APPs will be restricted by authority.

### 4.2. Opportunity and Challenge

mHealth care can provide personalized precision treatment and better people-centered care [38, 39]. This home-based rehabilitation model can better meet the needs of patients' autonomous rehabilitation management. At the same time, it can greatly improve the flexibility of rehabilitation management, reduce the medical burden, and better respond to special events such as COVID-19 [40].

The market prospect of developing intelligent auxiliary rehabilitation tools from the perspective of patients is very broad. This study provides reference for patients with different needs to choose appropriate intelligent auxiliary rehabilitation tools: the services provided by intelligent intervention and monitoring APPs are more humanized with easier instruction to operate, which is suitable for middle-aged and elderly patients who cannot master APP skillfully; the rehabilitation gaming APPs are more interesting, but the operation and use process is relatively complicated, which are more suitable for younger patients; and the angle measurement APPs require relevant rehabilitation theory knowledge and are more suitable for patients to utilize under the guidance of medical staff or with the assistance of family members. Meanwhile, through this research, detailed information in regard to APP instruction and operation modes of different types of rehabilitation software was obtained, which provides a basis for subsequent clinical promotion and utilization.

With the continuous deepening of intelligent medical reform, artificial intelligence algorithms have been widely used in auxiliary diagnostic methods of clinical medicine [41, 42]. APP has gradually become a new trend in medical diagnosis, treatment, prevention, and management. However, the clinical application of intelligent APP is still relatively immature, and the practicability and reliability need further verification. For example, in the collection of medical images, noise, image ambiguity, and complex clustering of multiview data [43–46]; permissions and pertinence and limitations of the crowd [47]; and cumbersome operation steps may lead to misoperation and privacy protection of information and data [48], etc. This study is conducive for medical staff to know the utilization of DRF-related APPs, understand high-quality intelligent rehabilitation APPs, and encourage the practical utilization inside and outside the hospital, so as to promote the expected effect of intelligent-assisted rehabilitation tools in clinical and patient home rehabilitation management.

## 5. Conclusions

With the development of the mHealth medical model, intelligent rehabilitation APPs and tools are gradually being used in clinical and patient independent health management. Through systematic evidence-based analysis based on literature and APP platforms, this study integrates different types of intelligent rehabilitation APPs that are suitable for DRF rehabilitation and explores its effects on the rehabilitation of such fractures from different perspectives. Although the evidence is limited, it can still be clearly shown that APP-based rehabilitation intervention, angle measurement, and monitoring management can all improve the effect of rehabilitation training and actively promote patients' self-rehabilitation management. This also enables clinical medical staff and DRF patients to make evidence-based choice according to the different characteristics and needs of APPs, which could meet the individual needs and improve the effectiveness of self-rehabilitation management. However, due to the restrictions of cost, geographic authority, and applicable population, a large number of APPs still lack effective evidence to support popularization. Therefore, in future research, large-scale user-centered clinical trials that will be added to evaluate the effectiveness and practicality of APPs are particularly important.

## Figures and Tables

**Table 1:** Brief information of the included studies.

Authors (year)	APP	System	Study type	Sample size	Method	Effect
Mohammad Reza Pour Ahmadi (2016)	G-Pro [9]	iOS Android	Cross-sectional observational study	38 men 32 women	The wrist flexion, extension, radial deviation, and ulnar deviation joint activity were measured by the universal goniometer and g-pro APP in iPhone 5, respectively.	The G-pro© APP has good to excellent reliability (ICC ≥ 0.73), and it is effective at the same time as the general goniometer (*r* ≥ 0.80) used to measure wrist ROM.
Jacob Modest BS (2018)	Compass [10]	iOS	Cross-sectional study	30 healthy 30 injured	The compass APP included in the conventional goniometer iPhone 5 was used to measure the joint activity during wrist flexion, extension, pronation, and supination, respectively.	Patients with wrist injuries can use this technology to perform self-measurements, and the measurements are comparable to standard UG ROM assessments performed by health providers.
Nuphar Lendner (2017)	Gyroscope [11]	iOS	Cross-sectional study	153	The flexion, extension, radial deviation, and ulnar deviation of the wrist were measured by common goniometer and free Gyroscope APP in iPhone 4, respectively.	It can improve compliance and is reliable and easy to use. Before using such an APP, it must be evaluated.
Robert H. Wellmon (2016)	Goniometer Records [12]	iOS Android	Descriptive validation study	NA	The research passed Goniometer Records of three smartphones; Goniometer Pro has two APPs and common goniometer. The inclinometer measures the joint activity of each standardized angle, respectively.	The app determines the error inherent in the measurement, which has nothing to do with patient factors and is attributed to the smartphone. The data shows that the installed APP can replace the conventional UG or inclinometer.
Susan Reid (2018)	DrGoniometer [13]	iOS	Clinical measurement study	30 fractured 30 healthy	Patients with DRF, and the forearm supination angle was measured by the universal goniometer and DrGoniometer, respectively. Healthy subjects were also measured by these two methods.	Whether it is a fractured forearm or a healthy forearm, the DrGoniometer and the universal goniometer are highly reliable (ICC range 0.74-0.88). DrGoniometer is an effective alternative tool.
Henriëtte A.W. Meijer (2019)	ReValidate! [14]	iOS	Cross-sectional study	45; 43	Patients recovering from wrist injury and professional wrist injury therapists completed a full-level game, respectively, and the experimenter finally scored the software, respectively.	ReValidate! is a beneficial and interesting experience for the rehabilitation of wrist fractures. It is tailored to patients and provides functional measurements for patients, which may become a very useful exercise tool in future rehabilitation.
Lori Algar OTD (2014)	Tilt Maze、Labyrinth [15]	iOS Android	NA	NA	NA	Skilled hand therapy can help recommend appropriate postures and encourage participation in therapeutic games, address specific client deficiencies, and reduce functional problems.
Xiaoxia Zhu (2019)	Rehabilitation assistant [16]	iOS Android	Randomized controlled trial	250; 250	The control group was treated with preintervention rehabilitation assistant APP, while the observation group was treated with postintervention rehabilitation assistant APP, and the patient satisfaction was compared	The health education effect and health education satisfaction of the rehabilitation assistant APP were higher than those before the intervention (*P* < 0.01 or *P* < 0.05).

## Data Availability

The data used to support the findings of this study are available from the corresponding author upon request.

## Supplementary Materials

Supplementary Materials The detailed information of 31 APPs related to the rehabilitation of distal radius fracture was contained in Supplementary Table 1, including the operation platform, user rating, download volume, payment method, and specific features description.
