# Supplementary material for: Intelligent Rehabilitation Assistance Tools for Distal Radius Fracture: A Systematic Review Based on Literatures and Mobile Application Stores
Source: Comput Math Methods Med. 2020 Sep 29;2020:7613569. doi: 10.1155/2020/7613569 (PMC7542482; doi:10.1155/2020/7613569)
Supplement: Supplementary Materials — The detailed information of 31 APPs related to the rehabilitation of distal radius fracture was contained in Supplementary Table 1, including the operation platform, user rating, download volume, payment method, and specific features description. [file 7613569.f1.docx]

Supplementary Table 1: Brief information of the included APPs from different platforms

| **APP** | **Language** | **Systems** | **Scores** | **Downloads** | **Payments** | **URL** | **Features description** |
| --- | --- | --- | --- | --- | --- | --- | --- |
| Healure | English | iOS/Android | 3.8 | 10,000+ | NA | https://play.google.com/store/apps/details?id=com.healure.painrecovery | 1. Free workout routines and Exercises;2. Video guidance;3. Personalized plan;4. Workouts for all levels 5. Workouts anytime, anywhere;6. Save money and time. |
| Physical Therapy Exercises | English | Android | 4.1 | 5,000+ | NA | https://play.google.com/store/apps/details?id=com.newWebApp.physicaltherapy&hl=zh_CN | 1.Slowly but surely you can move onto advanced physical therapy;2. Easy to understand language and easy to use interface;3. Easy navigation. |
| Rehand | English/French | iOS/Android | 3.8 | 5,000+ | NA | https://play.google.com/store/apps/details?id=com.healthinn.rehand | The exercise is conducted directly on the tablet screen. According to the performance, a series of music scores will be generated for you to observe the evolution. ReHand’s approach focused on trauma. In this way, through ReHand, you will be able to perform hand rehabilitation treatment in different trauma pathologies through specific exercises. |
| WristAlert | English | iOS/Android | 3.8 | 100+ | NA | https://play.google.com/store/apps/details?id=com.enflux.wrist | 1.Includes free unlimited access to the 10-day wrist carpal tunnel pain reduction curriculum;2. Damaging wrist posture alerts;3. Damaging wrist posture alerts;4. App & sensors provide carpal tunnel exercise for fast pain relief;5. Speed up recovery time and more. |
| Salaso | English | iOS/Android | 3.5 | 5,000+ | NA | https://play.google.com/store/apps/details?id=com.salaso.salaso | 1.Platform to Receive Videos of Physiotherapy Exercises Free Injury Prevention Exercise Videos; 2. Interactive Progress Tracking with Stats & Feedback Charts Personal Goal Setting;3. Logging Exercise Completion Monitoring Exercise Effort & Injury Pain Exercise Diary Reminder. |
| Fizioo | Danish/English | iOS/Android | 4.6 | 1,000+ | NA | 1. https://play.google.com/store/apps/details?id=io.cloudx9.fizioo | 1. Application for physiotherapy and rehabilitation services at home;2. Orthopedic rehabilitation、Neurological rehabilitation etc. |
| Rehab Guru Pro | English | iOS/Android | 4.2 | 5,000+ | NA | 1. https://play.google.com/store/apps/details?id=com.rehabguru.rehabguru | 1. Exercises created by fully qualified Exercise and Medical Professionals;2. Over 4500 exercises with 12000+ images;3. Clear instructional programmers sent via email;4. An improved professional service to your clients / patients;5. Live Search for exercises;6. Filter Search;7.Create your own exercise;8.Client Portal. |
| Physiotherapic Exercises | English | Android | 3.5 | 10,000+ | NA | 1. https://play.google.com/store/apps/details?id=com.HealthFitnessAndTutorials.PhysiotherapicExercises | 1. By using mechanical force and movements [Bio-mechanics or Kinesiology], Manual therapy, exercise therapy, and electrotherapy, remediates impairments and promotes mobility and function;2. Physiotherapy Exercises+Stretching Exercises. |
| Fisioterapia a tu alcance | Spanish | iOS/Android | 4.6 | 1000,000+ | NA | https://play.google.com/store/apps/details?id=com.goodbarber.fisioterapia | 1. Find videos of interest to you, find recently released videos, or go deep into physiotherapy by reading the best blogs on the entire Internet;2. Easy to understand and use exercises, instructions, and automated information without leaving your home;3. Provide personalized consultation and online consultation services to help you find solutions to injuries from a remote location. |
| MedBridge GO for Patients | English | iOS/Android | 4.8 | 100,000+ | NA | 1. https://play.google.com/store/apps/details?id=com.medbridgeed.hep.go | 1. Complete exercises as prescribed by your therapist;2. Set reminders, track overall progress, and view all of the Patient Education materials included by your therapist, from 3D models and explanatory exercise videos to clinician notes and PDF guides. |
| Simple Therapy | English | iOS | 4.9 | 1,00+ | NA | https://play.google.com/store/apps/details?id=com.simpletherapy.android.mhealth | 1. Easily Manage pain;2. Describe your pain and Simple Therapy will give you an evidence-based and clinically validated plan of care made just for your needs; 3. Manage full-body musculoskeletal pain by focusing on 18 areas of the body. |
| Physera | English | iOS/Android | 3.9 | 10,000+ | NA | 1. https://play.google.com/store/apps/details?id=com.healthcoda.physera | 1. Workouts targeting neck, knee, shoulder, hip, and other areas available too;2. Track your exercises and see how easy it is to build strength and flexibility;3. Workouts take as little as 5 minutes;4. Create your own. Build a custom workout using Physera’s free exercise library. |
| The Physio | English | Android | 4.8 | 1,000+ | NA | 1. https://play.google.com/store/apps/details?id=com.thephysioint | 1. Digitalizing health care for hassle-free experience;2. Dedicated Healthcare Professionals;3. Doctors Directory & Categorized view with 36 options;4. Interactive Clinic location on the map;5. Free transportation facility for appointments provided by Careem;6. Complete control and flexibility for Registered doctors with dedicated clinic Dashboard features;7. Customary Care & Feedback services for both Doctors & Patients 8. Scheduled In-Clinic or Home visits. |
| Sports rehabilitation training | Chinese | iOS/Android | 4.3 | 1,000+ | NA | https://apps.apple.com/cn/app/%E8%BF%90%E5%8A%A8%E5%BA%B7%E5%A4%8D%E8%AE%AD%E7%BB%83/id1480203025Store | 1. Through the "Customization" function, you can customize targeted sports programs and plans. This function can customize appropriate sports plans for yourself, and can also customize sports plans for others and share them with others;2. By scanning the app, the function can obtain the training plan customized by others and load it into your mobile phone to start training. |
| Help you | Chinese/English | iOS/Android | 3.1 | 100+ | NA | https://apps.apple.com/cn/app/%E5%B8%AE%E4%BD%A0-%E5%BA%B7%E5%A4%8D%E9%87%8F%E8%A7%92%E7%A5%9E%E5%99%A8/id1114498213 | 1. Through the vast number of rehabilitation patients to share their real rehabilitation experience, real and objective display of physical therapy and occupational therapy process of rehabilitation patients;2. In the process of recording, you can not only constantly motivate yourself, but also see the rehabilitation progress of others, and better cooperate with the rehabilitation therapist for rehabilitation treatment. |
| Sports rehabilitation | Chinese | iOS/Android | 3.9 | 10,000+ | NA | https://apps.apple.com/cn/app/%E5%B8%AE%E4%BD%A0-%E5%BA%B7%E5%A4%8D%E9%87%8F%E8%A7%92%E7%A5%9E%E5%99%A8/id1114498213 | 1. Understand common knowledge of prevention and treatment of clinical * common occupational-related diseases;2. Carry out preliminary evaluation on the current situation of users;3. The medical training part uses live demonstration and voice prompts to lead you to exercise rehabilitation treatment, and can formulate personalized treatment plan;4. Users can be reminded regularly for treatment. |
| OASIS Healthcare | Chinese/English | iOS/Android | 4.9 | 1000,000+ | NA | ps://apps.apple.com/cn/app/%E6%B3%93%E5%8D%8E%E5%8C%BB%E7%96%97-%E6%AD%A3%E8%A7%84%E5%8C%BB%E7%94%9F%E6%8A%A4%E5%A3%AB%E4%B8%8A%E9%97%A8%E6%8A%A4%E7%90%86%E5%B9%B3%E5%8F%B0/id990922589 | 1. Honghua Medical has a nationwide network of chain hospitals, clinics and home care services;2. The medical team is led by skilled expert nurses, who can go home for diagnosis and treatment according to your needs;3. It provides services such as international consultation, overseas medical care, green channel and health insurance. |
| AiXin rehabilitation | Chinese/English | iOS/Android | 3.3 | 1,000+ | NA | https://apps.apple.com/cn/app/%E8%89%BE%E6%96%B0%E5%BA%B7%E5%A4%8D/id1334111999 | 1. Users can make appointment registration for hospitals that have entered the platform, establish personal medical records, consult doctors free of charge, and provide professional medication and rehabilitation guidance;2. Provide perfect rehabilitation solutions for patients' needs and provide professional on-site rehabilitation and nursing services for special groups. |
| Postoperative rehabilitation | Chinese | iOS/Android | 5 | 100+ | NA | https://apps.apple.com/cn/app/%E6%9C%AF%E5%90%8E%E5%BA%B7%E5%A4%8D/id1404119954 | 1. Various surgical scenes, combined with wearable devices, and through vital signs, nutrition, exercise and other data, a quantitative postoperative evaluation scheme is designed, which is widely used in the field of postoperative accelerated rehabilitation;2. Help users understand their own physical condition and rehabilitation progress. |
| Joint doctor | Chinese | iOS/Android | 4.7 | 100+ | NA | https://apps.apple.com/cn/app/%E5%85%B3%E8%8A%82%E5%8C%BB%E7%94%9F%E6%82%A3%E8%80%85%E7%AB%AF/id1447482116 | 1. Preoperative access to disease-related knowledge, treatment information, hospitalization operation arrangements and other key information;2. Postoperative online and offline follow-up through voice, images and other means;3. Patients perform special and effective rehabilitation exercises according to personalized rehabilitation plans sent by competent doctors;4. Doctors monitor the rehabilitation status of patients at any time through app and supporting wearable equipment. |
| Gold nurse | Chinese | iOS/Android | 4 | 1000,000+ | NA | https://apps.apple.com/cn/app/%E9%87%91%E7%89%8C%E6%8A%A4%E5%A3%AB-%E4%BA%92%E8%81%94%E7%BD%91%E6%8A%A4%E7%90%86%E6%9C%8D%E5%8A%A1%E5%B9%B3%E5%8F%B0/id1075742311 | 1. Diverse service types to meet your daily care needs;2. Provide you with professional home care services 3. Establish private health files. |
| Angulus | Chinese/English | Android | 4.1 | 10,000+ | NA | https://play.google.com/store/apps/details?id=com.drinkplusplus.angle | 1.Save the measured image and send it by e-mail; 2. Load images/movies taken before or on the fly; 3.Drag points on an image to measure an arbitrary angle;4. Accurate measurement in 0.1-degree increment 5. Review/manage all record at once. |
| CJOrtho | French/English | iOS/Android | 4.6 | 5,000+ | NA | https://play.google.com/store/apps/details?id=fr.cjortho.app | 1. Classification most commonly used in traumatic orthopedic surgery;2. Tracking the patient's major clinical scores;3. Goniometer;4. A database for storing patient information and linking it with other tools in the application program. |
| Goniometer Records | English | Android | 3.9 | 50,000+ | NA | 1. https://play.google.com/store/apps/details?id=com.scriptlanes.goniometer | 1. Highly accurate digital Goniometer to measure angles in large joints, small joints and also of spine;2. Readings can be recorded with separate profile for each patient to keep a good record to check the sequential improvement in the range of motion at different follow up. |
| DrGoniometer | English | iOS | 3.0 | 1,000+ | US$19.99 | 1. https://apps.apple.com/cn/app/drgoniometer/id362356791 | 1. Enables the management of patient records, measurement of patient articulation angles and storage of all related information to build up historical data bases for each articulation and movement;2. Computer browse all Dr. Goniometer data and print any patient articulation record;3. Backup all Dr. Goniometer data on the computer;4. Use a previously created back-up to restore all Dr. Goniometer data on any iPhone;5. Export all measures in a Excel file through iTunes File Sharing, connecting your iPhone and PC through the USB cable. |
| PT-helper pro | English | iOS/Android | 4.4 | 1,000+ | US$2.99 | 1. https://play.google.com/store/apps/details?id=com.itransition.android.pthelper | 1. Animated exercise programs to lead you through your Favorite exercises;2. Multiple illustrations within each exercise to clearly show proper exercise form;3. Written exercise description;4. Timers for hold and recovery times to pace your exercises;5. Repetition and Set counters. |
| BlueJay Engage | English | iOS/Android | 4.2 | 1,000+ | NA | 1. https://play.google.com/store/apps/details?id=com.bluejayPT.engage | 1. Receive home exercise programs or HEP from your therapist right to your phone or iPad;2. Chat with your therapist about using multimedia messages;3. Log your progress to monitor pain and movement;4. Browse the video library to create your own treatment plan to address your pain. |
| Goniometer pro  (G-Pro) | English | iOS/Android | 3.4 | 1,000+ | NA | https://apps.apple.com/cn/app/%E9%87%8F%E8%A7%92%E5%99%A8/id646925503 | 1. Goniometer Pro can provide easy and accurate range of motion (ROM) readings;2. And for initial consultations, re-evaluations, and day to day office visits providing patients a way to see their progress. |
| Goniometer | English | iOS | 3.3 | 1,000+ | US$4.99 | 1. https://apps.apple.com/cn/app/getmyrom/id438534405 | 1. Just place your device against two surfaces in turn and Goniometer will give you the angle between them;2. In the protractor mode you can also determine the angle between lines on a plane: in order to do so align the edge of your device alternately against the sides of the angle;3. The accuracy of angles measurement is rather high: the error is only one degree! You'll appreciate the ability to save the measurement by taking a photo with the measured angle recorded on it. |
| GetMyRoM | English | iOS | 3.5 | 1,000+ | US$1.99 | 1. https://apps.apple.com/cn/app/goniometer-%E6%B5%8B%E8%A7%92%E5%99%A8/id406942245 | 1. Simple and intuitive user interface;2. Add Notes to your ROM measurements;3. Record Start/Stop and Total Joint Range of Motion;4. Select Side of Body、Select Joint、Select Motion;5. More accurate and easier to read than a standard bubble inclinometer;6. It includes the ability to save joint ROM by date and time. |
| PhysioNow | English | Android | 4.8 | 10,000+ | NA | 1. https://play.google.com/store/apps/details?id=com.physitrack.physiapp.physionow | 1. Be supported on your journey to recovery with 24/7 access to expert advice and tailored exercise programs;2. Carry out exercises in the comfort of your home with guided videos that can be downloaded and viewed at any time;3. Set yourself in-app reminders so that you always remember to complete your exercises;4. Record your progress and leave real-time feedback, allowing your physiotherapist to track your recovery and tailor your program as needed. |
